# Information asymmetry in the Kenyan medical laboratory sector

**DOI:** 10.1080/16549716.2021.1964172

**Published:** 2021-08-26

**Authors:** Felix Bahati, Mike English, Shahin Sayed, Susan Horton, Onyango Abel Odhiambo, Abdulatif A Samatar, Jacob McKnight

**Affiliations:** a Health Services Research Unit, KEMRI Wellcome Trust Research Programme, Nairobi, Kenya; b Nuffield Department of Medicine, University of Oxford, Oxford, United Kingdom; c Department of Pathology, Aga Khan University Hospital, Nairobi, Kenya; d School of Public Health and Health Systems, University of Waterloo, Waterloo, Canada; e Department of Regulatory Affairs, Kenya Medical Laboratory Technicians & Technologists Board, Nairobi, Kenya

**Keywords:** Essential diagnostics list, Kenya, diagnostics, health systems, pathology

## Abstract

**Background:**

Important information about medical laboratory providers is not readily available to all patients, clinicians nor regulators in Kenya. This study was conducted as part of a wider project aiming to improve access to high quality diagnostics by addressing information asymmetries in the Kenyan market for laboratory services.

**Objectives:**

The purpose of this study was to: 1) Gather pricing information for 49 common laboratory tests from medical laboratories in Nairobi, Kenya, noting where these prices were publicly available or withheld. 2) Assess patients’ knowledge of testing information including: turnaround time, price, and test availability.

**Method:**

This was a cross-sectional study where a mystery caller approach was used to survey 49 tests for turnaround time, price, and availability across 13 laboratories selected purposively. The mystery shopper survey was complemented by 251 patient exit interviews at two Kenyan hospitals to understand whether patients seeking laboratory tests in Nairobi had access to such information. All 251 patients were selected by convenience sampling.

**Results:**

We noted that 85% of the private laboratories did not disclose test prices and turnaround times to their patients. There was a wide range of prices on several key tests, with private in-facility laboratories charging an average test price of 468% of the average test price in public laboratories across all the 49 tests. We also found that many patients lacked key information regarding the tests they needed: 65% did not know the purpose of the test while 41% did not know the test price at all.

**Conclusion:**

Under the current system, patients have limited access to information regarding the key criteria required to make a rational decision. This has a significant impact on the quality, price, and turnaround time (TAT) offered by the medical laboratories that operate in this dysfunctional market.

## Background

Timely access to laboratory diagnosis is crucial to the provision of high quality health care [1]. This is especially important with the current epidemiological transition to a higher prevalence of non-communicable diseases [2,3] that require early laboratory detection for proper management and care. Barriers to accessing timely diagnosis have been reported in Sub-Saharan Africa (SSA) and other Low and Middle Income Countries (LMICs) [4]. These barriers include; insufficient human resource, inadequate infrastructure, poor quality, reagent stock outs and frequent equipment breakdowns leading to test unavailability [4,5]. Usually, patients are presented with a choice of seeking medical laboratory services in the public sector, private sector, or from faith-based organisations. As has been demonstrated for Kampala, a majority of the medical laboratory services in Nairobi are provided by the private sector [6,7]. Although private facilities, especially the private stand-alone laboratories, play a crucial role in helping patients access diagnostic tests that may not be available in some hospitals, the dependence on facilities external to public hospitals has resulted in disjointed health seeking journeys, thus undermining the concept of a ‘one stop shop’ for patients.

In some contexts, where patients are not covered by insurance and are paying out of pocket [8], they tend to be very sensitive to the price of laboratory testing. Patients with financial constraints are likely to seek diagnostic services from public laboratories where prices are relatively low, an indication that the Universal Health Coverage (UHC) idea is timely. Unfortunately, while the public sector is expected to offer the lowest costs, resource and capacity constraints often limit the tests available meaning that patients may need to seek laboratory testing outside of this sector [9]. Conversely, patients who are financially better off may use private laboratories where test prices are relatively higher because they believe the quality may also be higher. These customers are likely driven by the idea that ‘You get what you pay for’, but others have shown the failure of this logic in this field [7]. Additionally, convenience also plays an important role for patients seeking laboratory tests. Normally, patients and clinicians choose medical laboratories that are convenient to them in terms of accessibility and those that offer a shorter turnaround time [10].

Patients, like their clinicians, also expect high quality diagnostic laboratory services. According to WHO, laboratory quality is defined by the accuracy, reliability, and timeliness of the reported test results [11]. To achieve certificates of quality such as ISO 15,189 [12], clinical laboratories need to operate within a standard set of procedures and ensure an acceptable score on all the metrics or indicators of quality. Such indicators include well-calibrated laboratory equipment, skilled personnel, regular internal audits, proper document control processes, effective management of laboratory operations, proper reporting of test results, and clearly stipulated TurnAround Times (TATs)] [11,12]. These metrics are examined through quality assessment programs such as Internal Quality Control (IQC), Proficiency Testing (PT) and laboratory accreditation. Both IQC and PT are pathways to ISO 15,189 accreditation that provides an international standard on how a medical laboratory should be operated. Accreditation to ISO 15,189 is not mandatory for clinical laboratories in Kenya however, and is well beyond the financial reach of most laboratories.

In summary, research to date suggest that patients make choices based on test price, convenience and their own perception of quality [13]. These perceptions of quality and satisfaction among test seeking patients may include TAT, clear instructions during specimen collection, availability of the requested tests, fair cost of service and complete test results [14]. Classical economics [15] tells us that in a well-functioning market, laboratories would compete with each other on the basis of their performance in each of these categories. Patient-customers would encourage this competition by making rational choices based on good information and the subsequent competition for patients would ensure the health of the market. We set out to obtain preliminary data to explore information asymmetries in the laboratory sector of a major urban African setting. First, we surveyed test availability, and the willingness of major medical laboratories to share testing information on test prices and TATs. We then investigated patients’ knowledge of key testing information. We used a mystery caller approach to collect information on test availability, test prices and TATs from laboratories that did not provide this information publicly. We purposively surveyed those laboratories who have a bigger market share and many outlets meaning we collected information for 91 of the 522 laboratories registered in Nairobi at the time of the survey. This was followed by patient exit interviews within two major public hospitals in Nairobi city. We adopted a convenience sampling method to interview 251 patients who had been referred by clinicians and doctors to seek diagnostic tests from the laboratory sections of the two hospitals. Both sections of the research were conducted between March and June 2019. The findings of this study can inform efforts to improve functioning of the market for laboratory tests.

## Methods

### Ethics

This study was approved by the Aga Khan university Ethics Committee (Ref No: 2017/REC-103 (V6)).

It is regrettable that we were obliged to use a mystery shopper approach to gather test information for many of the laboratories. Such approaches do not allow for informed consent, and it would have been preferable to have communicated openly with the laboratory providers, but this option was not possible unfortunately. Anecdotal evidence would suggest that the laboratories withhold pricing information because they do not wish to compete on price. This issue should be a focus of further study, but for practical reasons during this preliminary research, we adopted the mystery shopper approach.

### Setting

This study was conducted in Nairobi, the capital city of Kenya. According to the 2019 census report, Nairobi has a population size of about 4.4 million people [16]. The medical laboratories in Nairobi can be classified as public and private with the latter representing a majority. The private medical laboratories are comprised of profit and not-for-profit private laboratories such as those owned by Non-Governmental Organizations (NGOs), of which the faith-based are one subgroup. Some of these privately owned laboratories are within a facility such as a pharmacy, while others are stand-alone laboratories. The laboratory services in Kenya are regulated by the Kenya Medical Laboratory Technicians and Technologists Board (KMLTTB). According to KMLTTB records, at least 522 medical laboratories had been licensed to operate in Nairobi at the time of study commencement. These consisted of 3 public, 12 faith-based and 507 private laboratories of which 505 were for profit while 2 of them were not-for-profit.

### Data collection

We collected primary data both on test prices and availability from laboratories as well as from patients seeking tests.

## Pricing survey

A list of all the licensed medical laboratories in Nairobi was obtained from the KMLTTB and purposive sampling was used to generate a list of the top medical laboratory service providers. In the purposive sampling approach, priority was given to laboratories that have a large share of the private clinical laboratory market, operated as chains and had operational licenses as at the time of the survey. We also included an academic (not-for-profit) laboratory, faith-based and public clinical laboratories. Our study sample was made up of 13 medical laboratories consisting of seven private laboratories, two faith-based, three public and one academic (not-for-profit) laboratory. The private laboratories were later categorized into stand-alone and ‘in-facility’ laboratories. One of the three public laboratories was attached to the National Referral Hospital (NRH). All seven private laboratories operated as part of large commercial chains. The seven chains represented by these seven laboratories have a total of 91 laboratories in their networks within Nairobi. This represents about 18% of the total number of the licensed private laboratories.

The cross-sectional survey was conducted between March and June 2019 to obtain testing information for each of the listed 13 laboratories. The information sought for every laboratory included test availability, test prices and the respective TAT. Most of the tests used in this survey were obtained from a list of the top 25 tests that Horton et al found to be the most common by volume across six different international hospital sites [1] (Appendix 1). We specified the tests with the sample type or analyte and this increased our test numbers from 25 to 49. For example, a test stated by Horton et al as Calcium was specified into Calcium ion, Calcium in urine and Calcium in serum. All 25 tests also appear in the WHO’s Essential Diagnostic List [17].

## Mystery caller approach

Save for one private stand-alone laboratory, most of the private and faith-based medical laboratories did not display their test price and TAT publicly to their customers. Although test prices and TATs among some public hospitals were not displayed publicly, patients who inquired were informed how much the test would cost and the stipulated TAT. The mystery caller approach was used to collect this information from the laboratories that did not display the information publicly [18–20]. Two trained research assistants made phone calls to the private and faith-based laboratories. During the call, the research assistants inquired about the test price, TAT and availability of any of the two tests picked from our list of 49 tests. For example, a research assistant would call pretending to be a patient that had been requested by a doctor to have a malaria smear and random blood sugar tests conducted. The research assistant would proceed to inquire if the laboratory had the capability to offer the tests at that moment, how much each of the tests would cost, and what the TAT of each of the tests would be. This was repeated to all the relevant laboratories on the same day and each of the research assistants would embark on another pair of tests the following day. The mystery callers specified the test name and the test sample during calls in order to avoid any ambiguity in the pricing and TAT information provided. Although the mystery calls were made to each of the central laboratories of a given chain, a confirmation of the price was attained by calling satellite laboratories linked to the laboratory chain within Nairobi and inquiring about the same. The information provided through the calls were recorded in notebooks and then entered into a Microsoft Excel spreadsheet after the call. All the satellite laboratories in Nairobi belonging to the same chain had similar test prices except one chain where test prices varied from one satellite to another within Nairobi city. Hence for this provider, only the prices reported from the central laboratory were used in the analysis. Quality assurance information regarding accreditation of these laboratories was obtained from the respective laboratory websites and confirmed by the publicly available databases of the Kenya Accreditation Services (KENAS) [21], South African National Accreditation System (SANAS) [22] and the College of American Pathologists (CAP) [23].

## Patient exit interviews

We sought to understand if patients had access to relevant information about the tests they needed and how the information they had informed their choices. To achieve this, convenience sampling was used to recruit patients seeking laboratory tests for face to face interviews as we did not want to cause long queues and unnecessary delays [24]. A semi-structured interviewer administered questionnaire was used. The study investigators sought permission to speak to patients seeking laboratory tests in two public hospitals in Nairobi. Public hospitals were chosen because most of the patients in Nairobi use public facilities for consultation and treatment. However, these public facilities tend not to have the required tests available dependably or the tests are not covered by public insurance.

We set up a station at the entrance of each of the two laboratories for two days at each facility and patients exiting all clinics including antenatal care with test request forms were directed to us by the clinicians. Data were collected in two sessions every day, the morning session and the afternoon session. At least 60 study participants of 18 years and above were targeted on each day of data collection. This was approximately 30% of all the patients seeking laboratory tests in their respective hospitals. Patients under 18 years of age were only recruited if they were accompanied by an adult who became the study respondent.

A semi-structured questionnaire was then used to obtain information from the patients and their guardians. For example, the patients were asked whether they knew the price of the test they wanted to request, its availability and the TAT. The tests requested were confirmed from the patients’ test request forms. Additionally, we also asked them if they considered test quality when seeking for laboratory tests, how they identified good quality laboratories and if they knew that poor quality tests could result in misdiagnosis. We later informed them of the exact test price, TAT and whether the laboratory in the same public facility was offering the test at that time. All the responses provided by the participants were entered into REDCap and sent to the server of KEMRI Wellcome Trust.

## Data management and analysis

**Figure 1.**
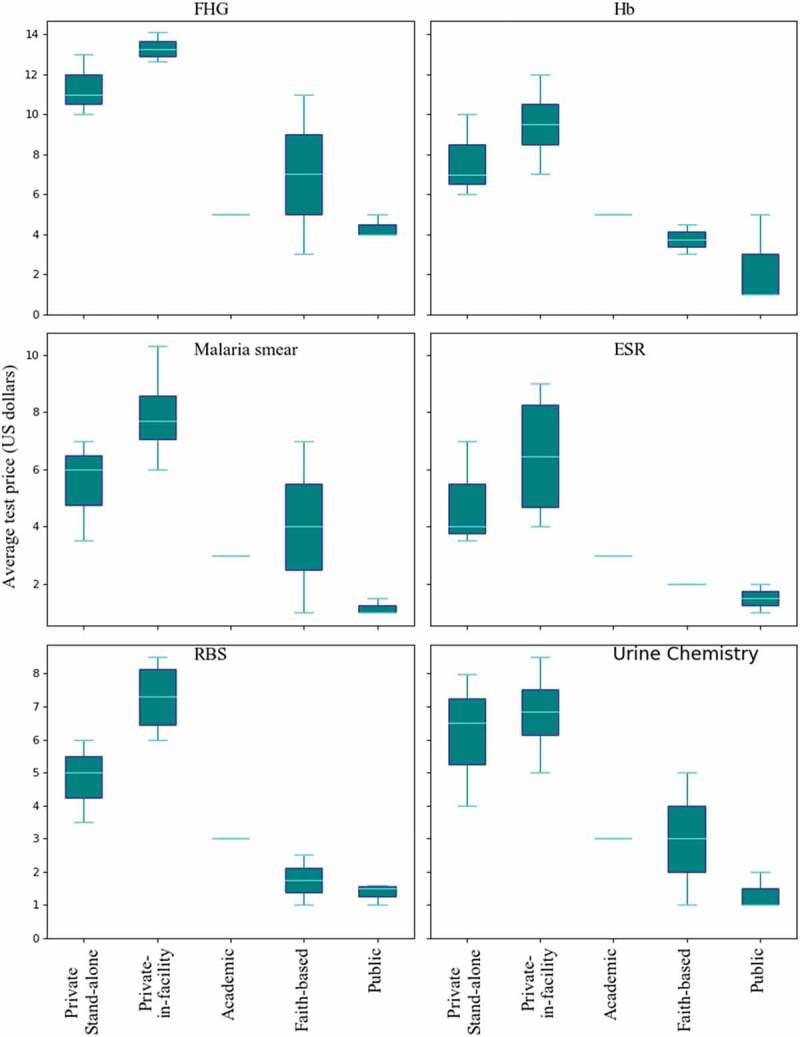
Comparison of the average test prices of the most common tests among clinical laboratories in Kenya, 2019. The clinical laboratories studied consisted of private stand-alone (N = 3), private-in-facility (N = 4), academic (N = 1), faith-based (N = 2) and public laboratories (N = 3). The tests include Full haemogram (FHG), Haemoglobin test using Hb meter (Hb), Malaria smear, Erythrocyte Sedimentation Rate (ESR), Random Blood Sugar (RBS), and Urine chemistry. These six tests were selected for this comparison based on the fact that each of the studied laboratories was able to offer them at the time of the survey. The test prices were converted from Kenyan shillings to USD at a rate of KSH 100 for 1 USD

Although data were collected from 251 patients seeking laboratory tests, a total of 6 respondents were excluded from the analysis due to incomplete data. The findings reported are therefore based on the responses provided by 245 study participants. The data were analysed both in Microsoft excel 2016 and R statistical software version 4.0.5 [25]. Microsoft excel 2016 was used to summarise the data as percentages, means, proportions and some of the graphical presentations. We used R statistical software to show the variations in average test prices of common tests presented in Figure 1. Test availability was calculated by counting the number of tests a given laboratory was able to offer of the total 49 tests and then converted to a percentage.

## Results

Pricing Survey: The study collected information on 49 tests within 13 central laboratories (described above). We noted that 85.7% of the private facilities and the two faith-based laboratories did not provide information on test price and TAT publicly to the patients. None of the three public facilities displayed their TATs publicly to their clients. However, two of these public laboratories displayed their test prices for their clients to refer and consider. Test availability was highest in stand-alone laboratories as they offered 95.9% of the tests on average, compared with 91.8% in private-in-facility laboratories and 51% in public laboratories. Test availability within public laboratories 8, 9 and 10 was 65.3%, 40.8% and 46.9% respectively (Table 1). In terms of quality assurance, all the private and faith-based laboratories surveyed had their central laboratories accredited to ISO 15,189 by at least one quality assurance provider (Table 1). Most of the accredited laboratories were accredited by KENAS although few others were accredited by SANAS and CAP.

**Table 1.** Test availability and publicly available information on price and turnaround time (TAT) of tests among clinical laboratories in Kenya, 2019

**Laboratories** **surveyed**	**Pricing information public**	**TAT information public**	**Number of 49 tests offered (%)**	**Main laboratory accredited**
Private-in-facility				
Laboratory 1	No	No	49 (100)	Yes
Laboratory 2	No	No	47 (95.9)	Yes
Laboratory 3	No	No	40 (81.6)	Yes
Laboratory 4	No	No	45 (91.8)	Yes
Private Stand-alone				
Laboratory 5	Yes	Yes	49 (100)	Yes
Laboratory 6	No	No	48 (97.6)	Yes
Laboratory 7	No	No	44 (89.8)	Yes
Public				
Laboratory 8	No	No	32 (65.3)	Yes
Laboratory 9	Yes	No	20 (40.8)	No
Laboratory 10	Yes	No	23 (46.9)	No
Faith-based				
Laboratory 11	No	No	40 (81.6)	Yes
Laboratory 12	No	No	39 (79.6)	Yes
Academic				
Laboratory 13	Yes	Yes	23 (46.9)	No

There was great variation in test prices among the laboratories. Mean prices across all 49 tests in stand-alone private laboratories were 300% of the mean in public laboratories and 468% in private in-facility laboratories compared to public. Public laboratories have the lowest mean test prices, with some tests such as HIV viral load and other tests for children under 5 years being free of charge. Malaria smear test had the largest price discrepancy with the private-in-facility laboratories charging up to 6 times more on average as compared to the public laboratories (Figure 1).

### Customer/patient questionnaire

**Figure 2.**
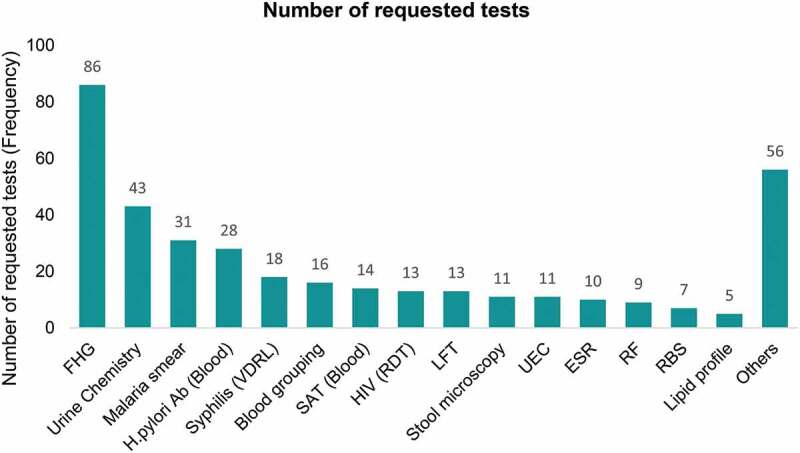
Number of the requested tests among clinical laboratories in Kenya. The tests include; Full haemogram (FHG), Urine chemistry, Malaria smear, Helicobactor pylori Antibodies in blood (H. pylori Ab (Blood)), Syphilis (Venereal Disease Research Laboratory test (VDRL)), Blood grouping, S. typhi Antigen Test in blood (SAT) Blood), Human Immunodeficiency Virus Rapid Detection Test (HIV(RDT)), Liver Function Test (LFT), Stool Microscopy, Urea Electrolytes and Creatinine panel (UEC), Erythrocyte Sedimentation Rate (ESR), Rheumatoid Factor (RF), Random Blood Sugar (RBS), Lipid profile and others. The total number of tests ordered was 371 (n = 371)

A total of 251 patients or their guardians were interviewed from the two public hospitals although our analysis was based on 245 of them who had complete data. Of the 245 respondents, 70.6% were female while 29.4% were male. The average age of the respondents was 33.7 years and 60.8% of them were employed. The overall number of tests requested was 371 as observed from the 245 test request forms. The highest number of tests noted on a single test request form were six and these included: Liver Function Tests, full haemogram (Complete Blood Count plus a smear), sickling test, Urine chemistry, Oral Glucose Tolerance Test and Syphilis test (VDRL). The full haemogram test, Urine chemistry and Malaria smears were the most frequently ordered tests at 86, 43 and 31 respectively as shown in Figure 2.

Of all the 245 study respondents, 64.9% did not know the purpose of the laboratory tests requested by the clinician. Only 86 participants attempted to state exactly what the laboratory test was for and out of these, 75.6% correctly determined the use of the laboratory test requested while 24.4% made an incorrect statement about the purpose of the test.

**Figure 3.**
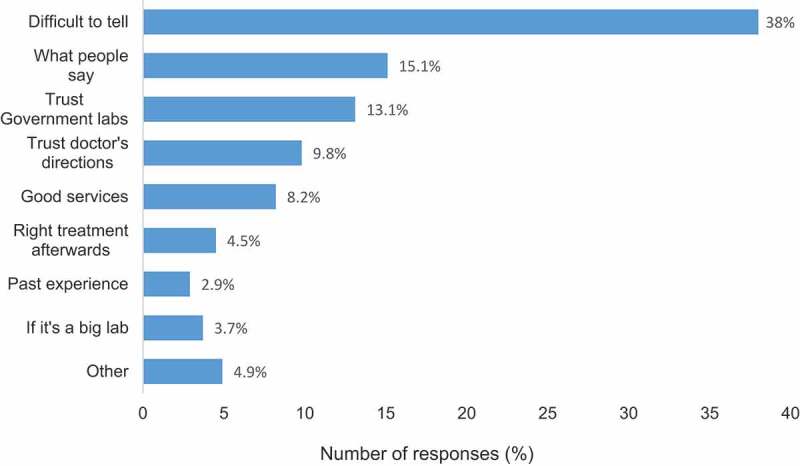
Criteria used by patients to identify good quality laboratories in Kenya. Each of the 245 respondents only selected one response resulting in 245 responses

The majority of patients had no information on the test price. Of the total respondents, 41.2% said that they did not know the price of the tests at all while a further 34.7% who attempted to estimate the test price underestimated it. On average, patients that attempted to guess the actual test price underestimated it by 41.5%. Although 59.6% of all the respondents mentioned that they valued quality when it comes to laboratory testing, 45.2% of those who valued quality agreed that it was difficult to identify a good quality laboratory. Generally, 38% of all the study respondents found it difficult distinguishing good quality laboratories from poor quality ones. Nonetheless, some patients (13.1%) trusted government laboratories as good quality laboratories while 15.1% others mentioned that they relied on reviews from other people such as friends and relatives as indicators of quality in medical laboratories. Almost 10% of the respondents trusted that doctors/clinicians always referred them to good quality laboratories (Figure 3).

**Figure 4.**
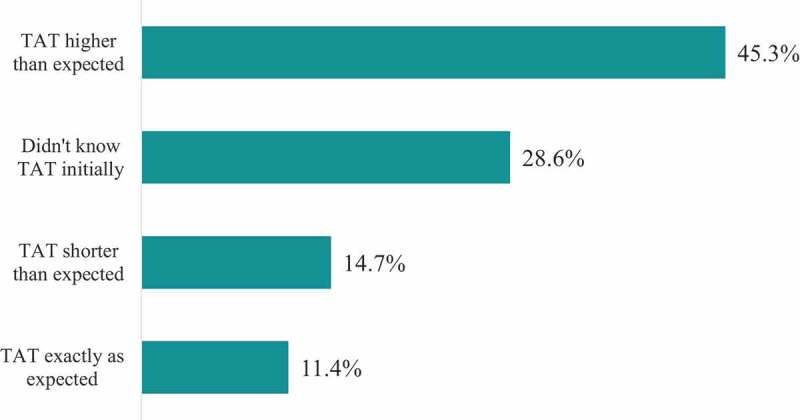
Patients’ feedback on TAT (n = 245). The expected turnaround time (TAT) is the waiting time that the patients guessed initially before we informed them the Actual TAT that the laboratories had provided to the study team

Based on the actual TAT information provided by the laboratories, the average TAT of the 371 tests requested by patients was two hours with a range of 1–72 hours. The longest turnaround time was 72 hours for a test requiring bacterial culture (high vaginal swab culture and sensitivity test). At least, 28% of the interviewed patients did not know how long they would wait to get their laboratory results. Almost half of the respondents underestimated TAT while 14.7% overestimated the TATs. Only 11.4% of the laboratory seeking patients correctly identified the TATs of their tests (Figure 4).

## Discussion

Our preliminary data revealed a significant lack of transparency in the medical laboratory market in Nairobi, especially in the private sector. While opacity on price is well noted in developed settings [26], we are not aware of similar findings for East Africa or other LMICs. In the USA, Mehrotra et al observed that although a significant proportion of health care seeking patients preferred to compare prices across providers before obtaining care, this information was not readily available [27]. About 85% of the private laboratories in our study did not provide information on price and TAT publicly to their potential clients. The reasons for this remain unclear, though anecdotally, it seems that fear of price competition in the private sector and highly variable supplier costs (partly driven by inflation) are some of the drivers. Presumably, this makes it difficult for patients to make informed decisions about which laboratories to visit when in need of diagnostic services. This opacity results in information asymmetry which, in turn, makes patients vulnerable to exploitation [28,29]. Although public laboratories have no restrictions regarding sharing of their pricing information and TAT, there is clearly a need for them to display the prices conveniently for patients. This could be the main reason as to why many patients interviewed did not know the exact prices of the tests that were readily available in public laboratories. Similarly, it is also possible that some of the patients who correctly identified the prices of tests could have obtained them from any relevant hospital department such as the payment section. Although the medical laboratory services in Kenya are regulated by KMLTTB, the KMLTTB has no control over the dissemination and display of test pricing, TAT or quality certificates. Generally, there is no legislation or policy in Kenya mandating all clinical laboratories to display their pricing information to their customers.

As the sole regulator of clinical laboratory services in Kenya, KMLTTB is mandated with five main responsibilities: indexing or examination of college students taking up careers in medical laboratory sciences; registration and licensing of all laboratory staff in Kenya; developing policy guidelines for the implementation of the Continuing Practice Development (CDP); and regulation of all In vitro Diagnostics (IVDs) intended for laboratory testing. Most importantly, KMLTTB regulates the conduct, practices and licensing of laboratory businesses in Kenya [30]. However, challenges such as financial constraints due to inadequate funding from the government have made the board focus more on licensing for revenue generation and it may be that this does not sustain the other mandates of the board. Also, overlapping roles between KMLTTB and other bodies such as Kenya Medical Practitioners and Dentist Board (KMPDB), Pharmacy and Poisons Board (PPB) and KENAS make it difficult for KMLTTB to accomplish all its roles effectively [30]. As part of its role in laboratory business regulation, the KMLTTB also enforces mandatory quality assurance through regular physical laboratory visits to ascertain whether the laboratories and their staff have active practising licenses and that the IVDs used are validated [31]. Voluntary laboratory quality assurance in Kenya is independently managed by KENAS [32] which is a separate entity from KMLTTB, with similar splits in responsibilities notable in other SSA countries and Latin America [33]. However, in high-income countries such as the USA, UK and China, the regulatory agencies are also involved in mandatory and voluntary quality assurance of medical laboratories [34,35]. Generally, most of the clinical laboratory regulatory agencies worldwide do not seem to have control over test prices and TATs. If the clinical laboratories in Kenya were to make testing information public, the regulator would more easily monitor the trends in test prices which could guide policies to ensure that patients have access to laboratory diagnostic services.

The average test price among private-in-facility laboratories was 468% of the average test price in public laboratories. Similar findings on test price variations have also been reported among private laboratories in Kampala, Zimbabwe and the USA [36–38]. The high test prices in these laboratories could be attributed to costs associated with maintaining quality assurance, but other contributing factors may be related to the quality of kits and equipment being used. Quite often, health insurers limit their clients to specific healthcare providers and under such restrictions, patients may not have alternatives even if prices are higher. These in-facility laboratories provide a one-stop shop unlike the stand-alone laboratories where patients have to move back and forth and themselves become responsible for conveying test information. Additionally, the relatively high test availability (91.8%) within the private-in-facility laboratories guarantees test accessibility to their patients, and this may allow these laboratories to charge a higher rate across all tests.

Slightly more than half of the participants interviewed (59.6%) said that they value quality when seeking for laboratory tests. Unfortunately, many of them find it difficult to differentiate between good and poor-quality laboratories. At least 13% of the interviewed patients said that they relied on government laboratories for quality tests while 10% mentioned that they trusted laboratories to which their clinicians or doctors referred them. Some clinicians have relationships with laboratories and are incentivized to send their patients to those laboratories while in other cases, they simply want a reliable result for their patient and for themselves. The fact, however, remains that government laboratories have limited test menus (47%-65%) mainly due to lack of proper equipment, reagent stock outs and inadequate personnel [5,39]. A similar finding on unavailability of essential tests has been reported among public hospitals in Ghana [40]. Under such circumstances, clinicians either treat empirically or make use of the few available tests to rule out some morbidities or justify a certain prescription. This could be the reason why the full haemogram test was frequently requested. It is possible that clinicians use this test as a primary means of checking for bacterial infection in blood [41]. Neither of the two public hospitals offered blood culture tests. Although full haemogram tests can help flag inflammation through monitoring of leucocytosis, not all inflammations are bacterial. This raises questions on the cost effectiveness and scientific use of laboratory testing [42,43]. Equipping public hospital laboratories with the necessary resources would therefore improve patient quality of care through test accessibility while protecting patients from high costs of private sector laboratory diagnoses. Test availability within the studied academic laboratory was low (46.9%), most likely because they have a major focus on academia and do not generally serve external, walk-in patients. Consequently, private laboratories provide a fallback for patients in need of laboratory testing. In some cases, patients may end up choosing laboratories based on pricing alone, but price is not a guide to quality [7,27] as even low quality testing laboratories could inflate their prices to maximize profit, and so there is a need to help both clinicians and patients to understand this.

Diagnostic testing is comprised of many processes that happen behind the closed doors of clinical laboratories. Evaluating quality and issuing certification requires both access and expertise. It would be desirable to educate patients and clinicians to seek services from laboratories that have certificates of participation in PT or verified quality accreditations. Laboratories taking part in such programs usually display these certificates publicly at the reception areas of their laboratories and on their websites. The main challenge, however, is that such certifications are not mandatory, and indeed, they come with costs that mean that most clinical laboratories do not have them. Although all the private laboratories that we surveyed were accredited, this is because we only included large private laboratories with bigger market share in this study. Unlike these few bigger laboratories, many of the small and medium private laboratories in Kenya are not accredited. There is an opportunity to make this information available to potential laboratory test seeking clients. Given the high literacy and smart phone ownership level in Nairobi city [44], one possibility is to use a digital marketplace app where patients can choose laboratories based on provided information such as test availability, test prices, TAT and quality certifications [45]. Besides helping patients, such a platform would also drive healthy competition whereby the quality certifications attained by each facility would more clearly be notable by patients and would become a more important point of comparison. We believe that this would be a good incentive to motivate the laboratories towards accreditation.

Regulation of laboratory test prices, especially in the private sector, is a complex issue and seemingly beyond the powers of most laboratory regulatory agencies. However, one possible way of ensuring accessibility to diagnostic testing is for the Kenyan government to introduce a policy ensuring that all public laboratories are well equipped to conduct all the essential diagnostic tests as set out by the WHO [17]. Such a policy would be timely, particularly given Kenya’s stated desire to achieve UHC [46]. This could reduce delays in finding desperately needed services by providing a one-stop-shop for patients seeking care in public facilities who tend to be poorer while also suffering from a higher disease burden [47]. Similarly, there is a need for continued monitoring of testing services in LMICs and this could be vastly improved by utilising existing resources such as the District Health Information System (DHIS2). Laboratory testing data are reported monthly from all general hospitals in Kenya including the private sector, but results are currently partial and low quality. Such data would be extremely useful in monitoring of laboratory test requests, test use and test accessibility of the primary essential tests. We suggest further research in this area to fully understand why most of the laboratories we purposively sampled did not share their test prices and TATs more publicly and how efforts to encourage this may improve overall access to laboratory diagnosis. Such findings could inform the MoH of possible actions to ensure transparency in the Kenyan medical laboratory market.

### Limitations

Although the mystery shopper approach is an accepted way of collecting withheld information, especially in the business sector, we did not obtain consent from the non-public laboratories from which we collected the pricing data and it would have been preferable to have had their full cooperation. However, this was the only viable approach of collecting the exact test prices charged to patients. The pricing data obtained in this survey were completely anonymized such that they cannot be linked back to the respective laboratories. Separately, we did not physically confirm from the laboratories whether they had the capacity to conduct the tests as informed during the mystery shopper call. This could have led to overreporting of test availability although it is rare for a laboratory to give false information regarding test availability to a prospective client whom they are expecting to visit their premises to have the test conducted. Most importantly, the findings reported in this study were summarized as proportions, percentages and means. The authors acknowledge that further statistical approaches would have been useful in drawing conclusive inferences from this study. For instance, inferential statistics comparing the average test prices across laboratory categories would confirm if the deviations in test prices were statistically significant. Nonetheless, it is clear from the calculated average test prices that different laboratory categories have different price ranges. It is also possible that our finding on low knowledge of test use among the participants could be attributed to literacy levels, but we are certain that cities such as Nairobi have the highest number of literates compared to non-urban settlements. Finally, this was a small study that aimed to collect preliminary data on test availability, test costs and patients’ access to such information within purposively selected facilities. The patients interviewed in the patient exit interviews within the two public hospitals were also selected based on convenience sampling to avoid delayed care consequently resulting to a small sample size. Therefore, the results obtained here may not be a representative of all the medical laboratories in Nairobi and patient responses were only gained from those using the public sector.

## Conclusion

Under the current system, patients have limited access to information regarding the key criteria required to make a rational decision. This has significant impact on the quality, price, and TAT offered by the medical laboratories that operate in this dysfunctional market. There is a clear need for all clinical laboratories to provide this vital information to their clients. Patients should also be given access to simpler metrics that they could use to distinguish poor and good quality laboratories. The opaque nature of testing information in the current clinical laboratory market in Nairobi limits access to high quality diagnostics and in turn, this limits the potential for higher quality medical care. There is an opportunity to enable this market to work better. In addition to more traditional means of working with the regulator and the ministry to encourage more open information in the market, we are excited by the prospect of digital health interventions. One possibility is to use a digital marketplace app, in the same way that has been done in other sectors where customers choose taxi drivers, second-hand sellers or holiday lettings.

## Paper context

**What was already known**

We know that patients struggle to access effective, timely and affordable diagnostics in LMICs and that this limits overall quality.

**What’s new**

We sought to understand whether patients in Nairobi have access to testing information such as test price, turnaround time, quality certifications and the related knowledge gap.

**What action needs to be taken**

Clinical laboratories, especially those in the private sector, need to make testing information readily available to the patients in order for them to make more rational choices.

## Appendix 1: Tests for the mystery shopper survey

**Table:**

Tests	
1	Complete blood count (CBC)
2	Urea, Electrolytes and creatinine (EUCs)
3	Creatinine (urine)
4	Urine chemistry
5	Malaria smear
6	Malaria Antigen test (RDT)
7	Random blood glucose
8	75 g Glucose Tolerance Test -OGTT (Normal)
9	Fasting glucose
10	Glucose tolerance, 2 hrs gestation
11	High-sensitivity C-Reactive Protein
12	C-Reactive Protein
13	Liver Function Tests
14	*Helicobacter pylori*, Antibodies in serum
15	*Helicobacter pylori*, Antigen in stool
16	Thyroid Function Panel
17	Urine Microscopy
18	Urine culture
19	HBA1c
20	Haemoglobin test (Hb meter)
21	HIV Screening Tests, ELISA
22	HIV viral load monitoring
23	HIV Rapid test
24	Erythrocyte Sedimentation Rate (ESR)
25	Pregnancy Test, serum (hCG, serum)
26	Pregnancy Test, RDT urine (hCG, Urine)
27	Calcium, serum
28	Calcium ionized
29	Calcium, Random urine
30	Blood culture
31	Syphilis Test, VDRL
32	Uric acid, Serum
33	Uric acid, urine
34	Magnesium, serum
35	Magnesium, urine
36	Troponin T
37	Troponin I
38	Lipid profile
39	TSH
40	Total bilirubin in serum
41	Direct bilirubin in serum
42	Bilirubin profile (Total and direct)
43	Pap smear
44	Total protein
45	Electrolytes
46	Phosphate Inorganic
47	Phosphate
48	Blood grouping and crossmatch
49	Crossmatch

## References

[1] Horton S, Fleming KA, Kuti M, et al. The top 25 laboratory tests by volume and revenue in five different Countries. Am J Clin Patho*l*. 2019;151(5):446–12. 10.1093/ajcp/aqy165 30535132

[2] Gaye B, Diop M, Narayanan K, et al. Epidemiological transition in morbidity : 10-year data from emergency consultations in Dakar, Senegal. BMJ Glob Health. 2019;4(4):1–7. 10.1136/bmjgh-2019-001396 PMC6666800 31406585

[3] Murray C, Barber R, Foreman K, et al. Global, regional, and national disability-adjusted life years (DALYs) for 306 diseases and injuries and healthy life expectancy (HALE) for 188 countries, 1990 – 2013: quantifying the epidemiological transition. Lancet. 2015;386(10009). 10.1016/S0140-6736(15)61340-X PMC4673910 26321261

[4] Wilson ML, Fleming KA, Kuti MA, et al. Access to pathology and laboratory medicine services : a crucial gap. Lancet. 2018;391:1927–1938. Available from. 29550029 10.1016/S0140-6736(18)30458-6

[5] Petti CA, Polage CR, Quinn TC, et al. Laboratory medicine in Africa : a barrier to effective health care. Clinical Infectious. 2006;42:377–382. 10.1086/499363 16392084

[6] Kenya National Bureau of Statistics. County statistical Abstract. 2015; Available from: https://www.knbs.or.ke/?p=3097&cp_county-statistical-abstracts=3

[7] Amukele T, Jones R, Elbireer A. Test cost and test accuracy in clinical laboratories in Kampala, Uganda. Am J Clin Pathol. 2018;149:522–529. 29659678 10.1093/ajcp/aqy017

[8] Salari P, Giorgio LD, Ilinca S, et al. The catastrophic and impoverishing effects of out- of- pocket healthcare payments in Kenya. BMJ Glob Health. 2018. 2019;4(6):1–13. 10.1136/bmjgh-2019-001809 PMC6882550 31803510

[9] Elbireer AM, Jackson JB, Sendagire H, et al. The good, the bad, and the unknown: quality of clinical laboratories in Kampala, Uganda. PLoS One. 2013;8:1–6. 10.1371/journal.pone.0064661 PMC3667826 23737993

[10] Hawkins RC. Laboratory turnaround time. Clin Biochem Rev. 2007;28:179–194. PMC2282400 18392122

[11] WHO. Quality management system handbook. 2011; Available from: https://www.who.int/ihr/publications/lqms_en.pdf

[12] ISO 15189:2012(en), medical laboratories — requirements for quality and competence [Internet]. [cited 2021 Jun 24]. Available from: https://www.iso.org/obp/ui/#iso:std:iso:15189:ed-3:v2:en

[13] Valenstein P. Laboratory Turnaround Time. Am J Clin Pathol. 1996;105(6):676–688. 10.1093/ajcp/105.6.676 8659441

[14] Hailu HA, Desale A, Yalew A, et al. Patients ’ satisfaction with clinical laboratory services in public hospitals in Ethiopia. BMC Health Services Research. 2020;9:1–9. 10.1186/s12913-019-4880-9 PMC6942306 31900148

[15] Hla M. The classical view of the economic problem. Economica. 2016;13:119–130.

[16] Kenya National Bureau of Statistics. 2019 Kenya population and housing census volume I: population by county and sub-county. [Internet]. Vol. I. 2019. Available from: https://www.knbs.or.ke/?wpdmpro=2019-kenya-population-and-housing-census-volume-i-population-by-county-and-sub-county

[17] WHO. Second WHO model list of essential in vitro diagnostics. 2019; available from: https://www.who.int/medical_devices/publications/Second_WHO_Model_List_of_Essential_In_Vitro_Diagnostics/en/10.1515/dx-2018-0035 29949510

[18] Rhodes KV, Miller FG. Simulated patient studies : an ethical analysis. The Milbank Quarterly. 2012;90:41756836. 10.1111/j.1468-0009.2012.00680.x PMC3530739 23216428

[19] Moriarty H, Mcleod D, Dowell A. Mystery shopping in health service evaluation. Br J Gen Pract. 2003;53(497):942–946. 14960218 PMC1314747

[20] Thi L, Oanh T, Nam DT, et al. Quantifying antimicrobial access and usage for paediatric diarrhoeal disease in an urban community setting in Asia. J Antimicrob Chemother.2018;73(9):2546–2554. 10.1093/jac/dky231 PMC6105870 29982636

[21] Medical laboratories – kenya accreditation services [Internet]. [cited 2021 Jun 6]. Available from: https://kenas.go.ke/cabs/medical-laboratories/

[22] South African national accreditation system | national, regional, global trust [Internet]. [cited 2021 Jun 6]. Available from: https://www.sanas.co.za/Pages/index.aspx

[23] Accredited laboratory and biorepository directory | college of American pathologists [Internet]. [cited 2021 Jun 6]. Available from: https://www.cap.org/laboratory-improvement/accreditation/accredited-laboratory-and-biorepository-directory/

[24] Etikan I, Musa SA, Alkassim RS. Comparison of convenience sampling and purposive sampling. American Journal of Theoretical and Applied Statistics. 2016;5:1–4.

[25] R: the R project for statistical computing [Internet]. [cited 2021 Jun 24]. Available from: https://www.r-project.org/

[26] Sutherland R. The effect of for-profit laboratories on the accountability, integration, and cost of Canadian health care services. Open Med. 2012;6:166–170. PMC3654513 23687532

[27] Ateev M, Katie MD, Anna DS, et al. Re: Americans support price shopping for health care, but few actually seek out price information. J Urol. 2018;199:884–885. 29642346 10.1016/j.juro.2018.01.029

[28] Christensen EW, Arnould RJ. The impact of asymmetric information and ownership on nursing home access. International Journal of Health Economics and Management. 2019;5:273–297. Available from. https://www.jstor.org/stable/25067728 10.1007/s10754-005-1796-1 16082519

[29] Das J, Holla A, Mohpal A, et al. Quality and accountability in health care delivery : audit-study evidence from primary care in India †. American Economic Review. 2016;106:3765–3799. 10.1257/aer.20151138 29553219

[30] Tsala B, Ali A, Onyango A Roadmap to validation and verification of IVDs in Kenya. 2021; Available from: https://www.who.int/medical_devices/global_forum/3rd_gfmd/IVDsKenya.pdf

[31] MoH. Kenya health workforce report: the status of healthcare professionals in Kenya, 2015. 2015; Available from: https://taskforce.org/wp-content/uploads/2019/09/KHWF_2017Report_Fullreport_042317-MR-comments.pdf

[32] KENAS. Accreditation. 2018;23. Available from: https://www.kenas.go.ke

[33] Mazziotta D. Accreditation of clinical laboratories in the Latin-American region. Clin Biochem [Internet]. 2009;42:309. Available from. 10.1016/j.clinbiochem.2008.09.026 19863936

[34] The Royal College of Pathologists. The regulatory landscape for pathology services. 2018;1–5.

[35] Kessler A. Laboratory quality regulations and accreditation standards in Germany. Clin Biochem [Internet]. 2009;42:315. Available from. 10.1016/j.clinbiochem.2008.09.031 19863941

[36] Amukele TK, Schroeder LF, Jackson JB. Most clinical laboratory testing in Kampala occurs in high-volume, high-quality laboratories or low-volume, low-quality laboratories. Am J Clin Pathol. 2015;143 (1):50–56. 10.1309/AJCPCYA54DWZQPQT 25511142

[37] Hsia RY, Antwi YA, Nath JP. Variation in charges for 10 common blood tests in California hospitals: a cross-sectional analysis. BMJ Open. 2014;4:e005482–e005482. 10.1136/bmjopen-2014-005482 PMC4139626 25127708

[38] Musarurwa C, Nyamayaro T, Mujaji WB, et al. Clinical laboratory test prices in Zimbabwe : a case of profiteering? CAJM. 2012;58:33–38. 26255327

[39] MoH. Harmonised kenya health facility assessment 2018 report. 2018.

[40] Ward CL, Guo MZ, Amukele TK, et al. Availability and prices of WHO essential diagnostics in laboratories in West Africa: a landscape survey of diagnostic testing in Northern Ghana. J Appl Lab Med. 2021;6:51–62. 33438734 10.1093/jalm/jfaa190

[41] Streit S, Frey P, Singer S, et al. Clinical and haematological predictors of antibiotic prescribing for acute cough in adults in Swiss practices - An observational study. BMC Fam Pract. 2015;16:4–9. 25655784 10.1186/s12875-015-0226-9 PMC4328046

[42] Bogavac-stanojevic N, Jelic-ivanovic Z. The cost-effective laboratory: implementation of economic evaluation of laboratory testing. J Med Biochem. 2017;36:238–242. 10.1515/jomb-2017-0036 PMC6287218 30568540

[43] Hernandez JS. Cost-effectiveness of laboratory testing. Arch Pathol Lab Med. 2003127 (4): 440–445. 10.5858/2003-127-0440-COLT 12683871

[44] DataReportal. Digital in Kenya: all the statistics you need in 2021 — datareportal – global digital insights [Internet]. [cited 2021 Jun 6]. Available from: https://datareportal.com/reports/digital-2021-kenya

[45] McKnight J, Fleming K. Will health marketplace apps improve laboratories? [Internet]. Indian J Cancer. Wolters Kluwer Medknow Publications; 2019;56:p. 291–292. [cited 2021 Jun 24]. Available from: https://www.indianjcancer.com/article.asp?=0019-509X;year=2019;volume=56;issue=4;spage=291;epage=292;aulast=McKnight 10.4103/ijc.IJC_673_19 31607694 10.4103/ijc.IJC_673_19

[46] Edwine B, Peter N, Di M Towards universal health coverage in Kenya: are we on the right path? 2019; Available from: https://kemri-wellcome.org/zp-content/uploads/2019/04/200-MEASURING-PROGRESS-TOWARDS-UNIVERSAL-HEALTHCARE-COVERAGE.pdf

[47] Kruk ME, Pate M. The lancet global health commission on high quality health systems 1 year on: progress on a global imperative. Lancet Glob Heal. 2020;8:e30–2. 10.1016/S2214-109X(19)30485-1 31839136

